# Gestural and Verbal Evidence of Conceptual Representation Differences in Blind and Sighted Individuals

**DOI:** 10.1111/cogs.70125

**Published:** 2025-10-13

**Authors:** Ezgi Mamus, Laura J. Speed, Gerardo Ortega, Asifa Majid, Aslı Özyürek

**Affiliations:** ^1^ Centre for Language Studies Radboud University; ^2^ Max Planck Institute for Psycholinguistics; ^3^ Department of English Language and Linguistics University of Birmingham; ^4^ Department of Experimental Psychology University of Oxford; ^5^ Donders Centre for Cognition Radboud University

**Keywords:** Silent gesture, Blindness, Feature listing, Manipulable objects, Non‐manipulable objects, Animals

## Abstract

This preregistered study examined whether visual experience influences conceptual representations by examining both gestural expression and feature listing. Gestures—mostly driven by analog mappings of visuospatial and motoric experiences onto the body—offer a unique window into conceptual representations and provide complementary information not offered by language‐based features, which have been the focus of previous work. Thirty congenitally or early blind and 30 sighted Turkish speakers produced silent gestures and features for concepts from semantic categories that differentially rely on experience in visual (non‐manipulable objects and animals) and motor (manipulable objects) information. Blind individuals were less likely than sighted individuals to produce gestures for non‐manipulable objects and animals, but not for manipulable objects. Overall, the tendency to use a particular gesture strategy for specific semantic categories was similar across groups. However, blind participants relied less on drawing and personification strategies depicting visuospatial aspects of concepts than sighted participants. Feature‐listing revealed that blind participants share considerable conceptual knowledge with sighted participants, but their understanding differs in fine‐grained details, particularly for animals. Thus, while concepts appear broadly similar in blind and sighted individuals, this study reveals nuanced differences, too, highlighting the intricate role of visual experience in conceptual representations.

## 1 Introduction

An increasingly prominent view of concepts is that conceptual representations can rely on both perceptual‐motor simulation and linguistic information (e.g., Bi, 2021; Calzavarini, 2023; Connell, 2019; Davis & Yee, 2021; Dove, 2022, 2023), so‐called hybrid theories of semantic representation which go beyond traditional embodied theories of language (e.g., Barsalou, 1999; Gallese & Lakoff, 2005; Pulvermüller, 1999). When conceptual representations engage perceptual‐motor simulation, visual information appears to be prominent (e.g., Chen, Zhao, Long, Lu, & Huang, 2019; Lynott, Connell, Brysbaert, Brand, & Carney, 2020; Repetto, Rodella, Conca, Santi, & Catricalà, 2023; Speed & Brybaert, 2021; Speed & Majid, 2017). The majority of concepts—everything from living things (e.g., animals) to manipulable objects (e.g., tools)—are strongly associated with vision (e.g., Barsalou, 1999; Humphreys & Forde, 2001; Lynott et al., 2020). This has led to an interest in the differential sensorimotor experience of congenitally blind individuals so as to explore the role of direct visual experience in the formation and organization of conceptual knowledge (e.g., Bedny & Saxe, 2012; Bi, 2021; Bottini et al., 2020; Connolly, Gleitman, & Thompson‐Schill, 2007; Hauptman, Liu, & Bedny, 2024; Landau & Gleitman, 1985; Shepard & Cooper, 1992; Wang, Men, Gao, Caramazza, & Bi, 2020). However, variations in methodology across the literature make it difficult to conclude the extent to which visual experience influences concepts. To circumvent this issue, here we test the structure of conceptual knowledge in congenital or early blindness using two complementary methods. This within‐subject, multimethod approach enables us to examine the contribution of sensorimotor experience to the use of conceptual representation across distinct contexts.

In this preregistered study, we examine whether and how the expression of conceptual representations of a range of objects with different sensorimotor affordances differs or is shared between blind and sighted individuals using two different paradigms. We examined how blind and sighted individuals use gestures without speaking (i.e., silent gestures) to depict concepts, as well as a more traditional method of studying conceptual representations by collecting written feature listings for the same concepts. These two paradigms emphasize distinct facets of concepts. Gestures are advantageous for depicting direct analog representations of visuospatial properties thanks to their affordances, although they are restricted by the limitations of the visual‐manual modality. On the other hand, feature listing is restricted to linguistic expression, but it can give insight into concepts across modalities. By using two different paradigms, we are able to obtain converging, as well as complementary, evidence to better understand the role of visual experience in conceptual representations.

Numerous studies have investigated blind people's conceptual representations using mainly language‐based measures (e.g., similarity judgments and feature listing), including for domains such as color (Connolly et al., 2007; Kim, Aheimer, Montané Manrara, & Bedny, 2021; Landau & Gleitman, 1985; Marmor, 1978; Saysani, Corballis, & Corballis, 2018, 2021; Shepard & Cooper, 1992; Wang et al., 2020), visual metaphors (Minervino, Martín, Tavernini, & Trench, 2018), other visual properties, such as light emission (Bedny, Koster‐Hale, Elli, Yazzolino, & Saxe, 2019; Landau & Gleitman, 1985; Lenci, Baroni, Cazzolli, & Marotta, 2013), as well as animals (Kim, Elli, & Bedny, 2019; Tian et al., 2024), and various concrete and abstract concepts from different semantic classes (e.g., Bi, Wang, & Caramazza, 2016; Crollen & Collignon, 2020; Kanjlia, Lane, Feigenson, & Bedny, 2016; Lenci et al., 2013; Striem‐Amit, Wang, Bi, & Caramazza, 2018; Xu et al., 2023). The results of these studies suggest that blind people acquire considerable knowledge about concepts through indirect experience derived from language—such as learning the colors of objects and the meaning of vision verbs like *look* and *see* (e.g., Bi, 2021; Campbell, Casillas, & Bergelson, 2024; Landau & Gleitman, 1985). Many studies report no differences between blind and sighted people's semantic judgments (Bedny et al., 2019; Kim et al., 2021; Landau & Gleitman, 1985; Lewis, Zettersten, & Lupyan, 2019; Marmor, 1978; Ostarek, van Paridon, & Montero‐Melis, 2019; Saysani et al., 2018, 2021), suggesting the absence of vision has no demonstrable effect on conceptual representations, even for concepts that are primarily related to vision. This supports the proposal from amodal theories of conceptual representation that sensory information can be learned from language alone (see Bedny & Saxe, 2012, for a review). Accordingly, blind people could represent visual content in the same way that sighted people do.

Other studies, however, have revealed qualitative differences in the use of conceptual knowledge between blind and sighted people (Connolly et al., 2007; Kim et al., 2019; Lenci et al., 2013; Marques, 2010; Shepard & Cooper, 1992). In a feature‐listing study, Lenci et al. (2013) asked blind and sighted people to define concrete and abstract concepts by their core features. For concrete concepts (e.g., *pencil*), blind people reported fewer perceptual features (e.g., *being cylindrical*) but more contextual features (e.g., *paper*) than sighted people. More recently, Kim et al. (2019) found that blind people rely more on taxonomic knowledge than sighted people to reason about animal appearance, including size, shape, and color. They suggested that blind people have broadly similar categories to sighted people but differ in fine‐grained details, especially in properties that cannot be explored non‐visually (through touch, for example). Separately, Connolly et al. (2007) showed that despite knowing color information (e.g., apples are red), blind people—unlike sighted people—do not use color information to make similarity judgments about broader categories, such as fruits and vegetables. Together, these studies suggest that some visual features of concepts are not salient in the concepts of blind people, so that knowledge acquired only through language is not utilized in the same way. This implies that visual experience can affect the organization of conceptual knowledge.

The findings outlined above may reflect variation in research paradigms. Thus, the similarities and differences observed in the use of conceptual knowledge between blind and sighted people might be specific to particular tasks and not necessarily portray the complete picture. Conceptual representations can be flexibly used based on various factors specific to an individual's experience, a particular moment in time, and current task goals (e.g., Barsalou, 2016; Casasanto & Lupyan, 2015; Connell & Lynott, 2014; Dove, 2023; Pexman, 2019; Yee & Thompson‐Schill, 2016). It is, therefore, essential to use diverse contexts to gain a deeper and more complete understanding of conceptual representations. Thus, the present study aims to offer a different perspective on conceptual representations by examining their visual‐manual mappings onto the body through gestures of blind and sighted individuals for objects from different semantic categories. At the same time, we use the feature listing paradigm to compare verbal expressions of conceptual representations with their visual‐manual expressions.

It is now well‐established that gestures produced during speech or when no speech is allowed (i.e., silent gesture) provide unique insight into conceptual representations. Gestures are thought to arise from sensorimotor simulations underlying concepts and are driven by mappings of visuospatial and motoric experiences with objects (e.g., Beilock & Goldin‐Meadow, 2010; Cook & Tanenhaus, 2009; Goldin‐Meadow & Beilock, 2010; Hostetter & Alibali, 2008, 2019; Pouw, Wassenburg, Hostetter, de Koning, & Paas, 2020). These experiences may not be fully accessible through language‐based measures because some visual aspects of concepts—such as their particular shape or motor affordances—may be difficult to verbalize or seem redundant (Landau & Jackendoff, 1993; Levinson & Holler, 2014). Thus, they are less likely to appear in feature listings.

**Fig. 1:**
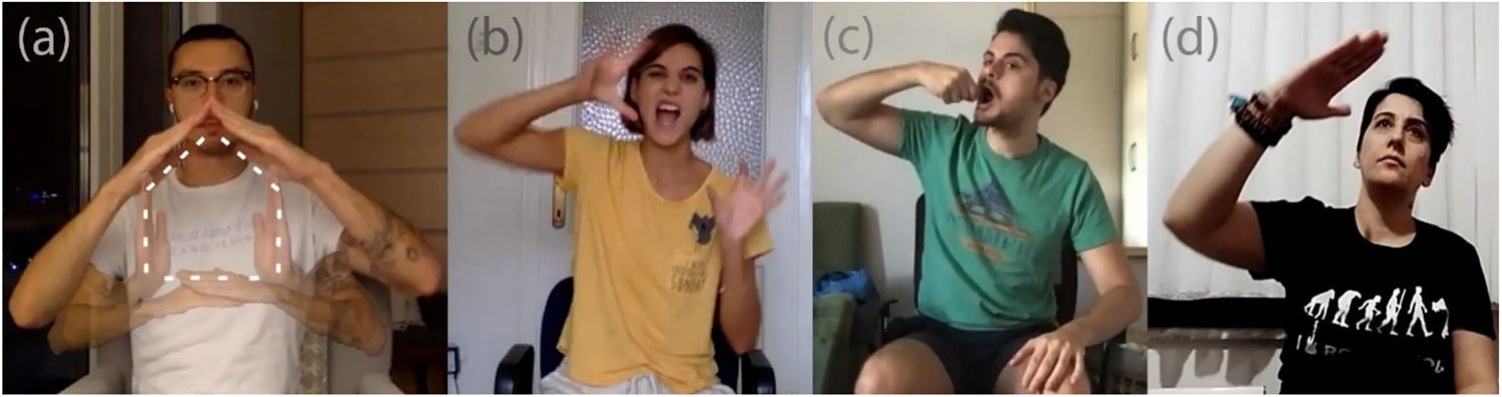
Different strategies in silent gestures are used: (a) drawing for *shed*, (b) personification for *lion*, (c) acting for *spoon*, and (d) representing for *plane*, from left to right.

Research has shown that concepts with different underlying sensorimotor associations can affect gestures in different ways. Studies with sighted individuals have examined the gestural representation of single semantic concepts (e.g., objects) in co‐speech (Masson‐Carro, Goudbeek, & Krahmer, 2016, 2017) or silent gestures (Brentari, Renzo, Keane, & Volterra, 2015; Ortega & Özyürek, 2020a, 2020b; Padden, Hwang, Lepic, & Seegers, 2015; van Nispen, van de Sandt‐Koenderman, & Krahmer, 2017) and found that concepts that trigger motor experience—such as manipulable objects, like tools—result in an acting strategy (i.e., the reenactment of a bodily action with or without an object). Conversely, when there are limited ways to manipulate referents, such as non‐manipulable objects and animals, sighted people tend to depict their visual appearance through a drawing strategy (i.e., tracing the outline of an object) or a personification strategy (i.e., embodiment of a non‐human entity by mapping its movement onto one's body), respectively. In addition, a representing strategy (i.e., hands representing the partial or full form of an object) is also used across semantic categories, albeit less frequently (see Fig. 1 for examples). Notably, similar strategies for different semantic categories are observed across communities (e.g., Dutch and Mexican; Ortega & Özyürek, 2020b).

To explain these systematic patterns, van Nispen et al. (2017), building on Taub's (2001) model, proposed that in silent gesture tasks, people select salient features of their mental representations that fit the constraints of the visual‐manual expression. Because of these constraints, some features—such as color—are more difficult to express in gesture, even when they are salient (though signers can make use of a range of iconic strategies to express colors; see Byun, Roberts, De Vos, Zeshan, & Levinson, 2022). Thus, people tend to rely on action‐based features (e.g., pouring from a bottle) or perception‐based features (e.g., the shape of a bottle). Motor experiences are particularly salient for manipulable objects and are readily expressible through the acting strategy. For distal or large objects, such as animals and non‐manipulable objects, due to the limited motor experience, visual experience plays a greater role in supporting the use of drawing and personification strategies (Ortega & Özyürek, 2020a; van Nispen et al., 2017).

As different sensorimotor experiences shape gestural forms for concepts, gestures offer an interesting perspective into blind and sighted people's conceptual representations through their analog expressions of underlying sensorimotor experiences. The few previous studies that have investigated the gestures of blind people have predominantly focused on spatial event representations, often addressing this from a cross‐linguistic perspective (Iverson, 1999; Iverson & Goldin‐Meadow, 1997; Mamus, Speed, Rissman, Majid, & Özyürek, 2023; Özçalışkan, Lucero, & Goldin‐Meadow, 2016, 2018). To our knowledge, no study has investigated the representations of object concepts by comparing the gestures of blind and sighted individuals. Using silent gestures and feature listings, we investigated whether the structure of conceptual representations differs between blind and sighted people for concepts from three semantic categories (i.e., manipulable objects, non‐manipulable objects, and animals) that are known to have different sensorimotor affordances (e.g., Damasio, Grabowski, Tranel, Hichwa, & Damasio, 1996; Farah & McClelland, 1991; Gainotti, Silveri, Daniel, & Giustolisi, 1995; Warrington & McCarthy, 1987; Warrington & Shallice, 1984; Yee, Chrysikou, Hoffman, & Thompson‐Schill, 2013).

If visuospatial and motor experience play a role in conceptual knowledge (Connell, 2019; Davis & Yee, 2021; Dove, 2023; Hostetter & Alibali, 2008; Ortega & Özyürek, 2020a; van Nispen et al., 2017), then there should be fewer gestures in the blind than sighted individuals for concepts that rely more on visual—that is, non‐manipulable objects and animals—than motor information, that is, manipulable objects. That is, there should be an interaction between the visual experience of participants and the semantic category they gesture. Specifically, there should not be a difference between blind and sighted participants in the frequency of gestures for manipulable object concepts, as the acting strategy (the preferred strategy for this category among sighted people) relies on motor experiences with objects, which is available to both blind and sighted people. However, there should be fewer gestures for non‐manipulable objects and animal concepts using drawing and personification strategies, respectively, as these depend on visual experience. Finally, there were no specific predictions for the representing strategy as it is not typically used for a particular semantic category (Ortega & Özyürek, 2020b).

Turning to feature listing, the strongest version of embodied account of meaning (e.g., Barsalou, 1999; Gallese & Lakoff, 2005; Pulvermüller, 1999) would predict differences in feature listing, such as the production of fewer perceptual features (e.g., related to color, appearance, and so on) by blind people. Conversely, blind participants may report more non‐perceptual features that are modality‐independent (e.g., functional, taxonomic, and encyclopedic information) compared to sighted participants, or, alternatively, both groups could perform similarly on these features (Bedny & Caramazza, 2011). Specifically, according to embodied theories, differences between blind and sighted participants should be larger for non‐manipulable objects and animals than for manipulable objects, as blind participants have richer perceptual experience with manipulable objects than with nonmanipulable objects and animals. Earlier empirical evidence supports these predictions by showing that blind people reported fewer perceptual but more contextual features for concrete objects than sighted people (Lenci et al., 2013). In our preregistration, we did not make specific predictions about different types of perceptual features, such as shape or sound, but planned to conduct exploratory analyses using data‐driven categories.

## 2 Method

### 2.1 Participants

As preregistered (available at https://osf.io/57qvn), 30 congenitally or early blind (11 Female, *M* = 32 years, *SD* = 14.14, range = 19–52) and 30 sighted (14 Female, *M* = 34 years, *SD* = 10.21, range = 18–57) native Turkish speakers were recruited online. At the time of testing, 18 blind participants had light perception, and 12 had total blindness (see Table S1 for detailed characteristics of the blind participants). All Supplementary Documents are available at https://osf.io/6j7xr. We aimed to match the 30 sighted participants, who had normal or corrected‐to‐normal vision, to the blind participants in terms of age, gender, and education. While the groups were not perfectly matched, there were no significant differences in age, *t*(57.8) = 0.08, *p* = .94, or education, *t*(56.8) = −1.07, *p* = .29. They all were paid the equivalent of €10 in Turkish Lira for their participation and provided online informed consent approved by the IRB committee of Radboud University.

### 2.2 Stimuli

There were 60 experimental items in total, 20 per semantic category: manipulable objects (e.g., spoon), non‐manipulable objects (e.g., bridge), animals (e.g., dog), and 4 practice items (i.e., ice cream, tree, book, and penguin, respectively). The item “castle” in the non‐manipulable object category had to be removed in the final analyses, as Turkish *kale* is a homonym of “goal,” and participants did not interpret the intended meaning. This left 19 non‐manipulable object words.

We initially selected 239 words for manipulable and non‐manipulable objects and animals from the silent gesture database produced by Ortega and Özyürek (2020a), other gesture studies (Masson‐Carro et al., 2016, 2017; van Nispen et al., 2017), and neuroimaging studies of semantic concepts (He et al., 2013; Peelen et al., 2013). In order to select 60 out of 239 items, we collected sensorimotor ratings from a separate group of Turkish sighted participants following Lynott et al. (2020). Participants rated each word on a scale from 1 to 5 on the extent to which a particular concept is experienced through six different sensory modalities (touch, hearing, smell, taste, sight, and introception) and five different action affordances (by performing an action with mouth/throat, hand/arm, foot/leg, head, and torso). The aim was to use touch and hand action ratings to operationalize manipulable versus non‐manipulable objects. We divided the 239 words into three lists, and each participant rated only one list online via Qualtrics (2022, Qualtrics, Provo, UT). Each word was rated by at least 21 participants, with a maximum of 33 participants.

To select the final set of words, we used sensorimotor ratings and word frequency information. First, we chose items with word frequency between 0 and 300 in 1 million words from various written Turkish texts (Göz, 2003). Then, we chose 20 manipulable objects with the highest rating of tactile and hand action experience and 20 non‐manipulable objects with the lowest rating of tactile and hand action experience. We replaced an item if a word (e.g., *fork* or *sailboat*) was semantically too similar to other selected words (e.g., *spoon* or *ship*). We did this to increase gesture diversity because we would expect similar gestures for objects such as spoon–fork and ship–sailboat. Furthermore, we replaced the items that we thought would be difficult to express by gesture—such as music and electricity. Finally, we chose 20 animals that had comparable word frequency with the manipulable and non‐manipulable objects. A one‐way ANOVA showed no difference in word frequency across semantic categories, *F*(2,57) = .039, *p* = .96. See Supplementary S1 for the list of 60 words with average word frequency and modality ratings. Audio files were recorded by a female Turkish speaker to serve as a prompt to both blind and sighted participants. The audio files of stimuli are available at OSF.

### 2.3 Procedure

Participants filled out an online informed consent document containing information about the experiment and data sharing, which was sent to participants as online forms via Qualtrics. Participants first performed the silent gesture task, followed immediately by the feature listing task without taking a break longer than 10 min. Participants also filled out a demographic questionnaire—including questions about blindness history for blind participants.

#### 2.3.1 Silent gesture task

The Zoom video platform was used to test participants due to COVID‐19 regulations. The experimenter recorded the session through the screen recording feature. Participants were presented with prerecorded spoken words and asked to explain each word within 6 s by using only gestures, facial mimicry, and body movements without speaking or leaving their seats. The instructions were adapted from Ortega and Özyürek (2020a)—see Supplementary S2 for the full instructions. They were also instructed not to point at objects around them during the task. For instance, if they heard the word *floor* they were not allowed to point to the floor in the room. Participants were told that another participant would watch their gestures later and guess their meaning. To determine an appropriate duration, we piloted 4‐, 6‐, and 8‐s windows and found that 4 s was too short and 8 s was too long, leading to repeated gestures. Thus, we chose 6 s to balance clarity and time pressure.

Each trial began with a 1‐s beep to indicate the upcoming trial, and then participants heard the audio recording of each experimental item twice. Following the second repetition, participants had 6 s to provide a gesture. After 6 s, they heard a 1‐s beep to indicate the end of the trial and the initiation of the next trial. Participants performed four practice trials in the same order, with words not included in the experiment. Experimental trials were presented in a different randomized order for each participant. At the end of the task, we asked participants to gesture again for any trials they had skipped without a time limit, so that they could take the time needed to think before making a second attempt to produce a gesture. The aim of this was to confirm that when a trial was skipped, it was not merely due to time pressure but rather because participants were unable to produce a gesture for a specific word. The results showed that both blind and sighted participants could produce gestures for some of the previously skipped trials on the second attempt, with no significant difference between the groups, χ^2^ (1) = 0.80, *p* = .37. This indicates that although some of the skipped trials might have been due to time pressure, this holds true for both participant groups, and blind participants did not need more time than sighted participants. These second attempts were not included in the analyses (see Supplementary S3 for the model summaries and the relevant plot). Each session lasted approximately 15 min.

#### 2.3.2 Feature listing task

In this task, participants were asked to list at least five features that are typically true of each word that they heard in the silent gesture task. The instructions were adapted from Papies et al. (2020) (see Supplementary S4 for the full instructions). Participants were informed that they would have 1 min to list features in a written form to describe the characteristics of each concept. Two examples (i.e., *blender* and *sun*) were provided. On each trial, participants were given a summary of the instructions. This questionnaire was administered via Qualtrics. The questionnaire lasted around 60 min.

### 2.4 Coding

#### 2.4.1 Gesture coding

Trials were annotated and coded by native Turkish speakers using ELAN (Wittenburg, Brugman, Russel, Klassmann, & Sloetjes, 2006). We first segmented each meaningful gesture for each word. Repetition of the same gesture was disregarded. Next, each gesture was classified, following Ortega and Özyürek (2020a), according to the strategy used (i.e., drawing, personification, acting, representing, and pointing).

Two people coded the strategies as either (a) drawing, if the gesture traced the outline of a concept by hand or index finger, (b) personification, if the gesturer embodied a non‐human animate entity or object by mapping its movement onto body, (c) acting, if the gesture imitated a bodily action with or without an object, (d) representing, if the hands represented the partial or full form of a concept, or (e) pointing^1^ if the gesturer pointed at an imaginary object (see Supplementary S5 for our coding scheme with examples). There were cases in which multiple gestures were observed in one trial, either sequentially or simultaneously (see Table S3). For example, to depict the concept *cat*, a sighted participant depicted the ears of a cat by placing two fingers on their heads (representing strategy) while enacting a cat meowing with the mouth movements (personification strategy). As these strategies were combined in one gesture simultaneously (not consecutively), this was coded as “double strategy” (see Supplementary S7 for additional information).

For how many gestures were present in each trial, the reliability of coding was assessed using the Intraclass Correlation Coefficient (ICC; Koo & Li, 2016). ICC was .94, indicating excellent inter‐rater reliability. For coding of strategy, Cohen's Kappa (Cohen, 1968) was used. Unweighted Cohen's Kappa was *κ* = .89 and weighted was *κ* = .91, indicating almost perfect agreement between raters (Landis & Koch, 1977).

#### 2.4.2 Feature coding

The features were first classified as perceptual or non‐perceptual, using adapted definitions from earlier feature norming studies (e.g., McRae, Cree, Seidenberg, & Mcnorgan, 2005; Vinson & Vigliocco, 2008). Perceptual features capture information gained through a primary sensory channel and can depict size, shape, appearance, sounds, body parts (e.g., *has 4 legs, has a tail*), object parts (e.g., *strap, with a handle*), kinematic information (e.g., *runs fast*), among other things. Perceptual features were later classified into different types: (i) visual‐only properties, such as color, appearance, and other visual properties (e.g., *shiny*, *bright*); (ii) multimodal properties (i.e., perceivable through vision and touch), such as magnitude (e.g., related to size such as *tall*, *big*), shape, and texture; (iii) non‐visual properties, such as sound, taste, smell, temperature; (iv) parts; and (v) kinematic movement.^2^

Non‐perceptual features could be of different types: (i) functional—how an object is used or its purpose (e.g., *used for writing*); (ii) taxonomic—including superordinate categories (e.g., *animal*, *tool*); (iii) encyclopedic—referring to object substance (e.g., *plastic*); (iv) animals’ habitat and diet (*carnivore*); (v) object location (e.g., *found in houses*); or (vi) other for non‐classified features (see Supplementary S8 for full coding scheme). A native Turkish speaker coded all the data. A second native speaker coded 50% of the features for reliability analysis. Weighted Cohen's Kappa was *κ* = .90 for type of feature (perceptual, non‐perceptual), which indicates almost perfect agreement (Landis & Koch, 1977).

## 3 Results

Following the preregistration, we analyzed the data using linear mixed‐effects and generalized linear mixed‐effects regression models (Baayen, Davidson, & Bates, 2008) with the fixed factor of visual status (blind or sighted), fixed factor of semantic category (manipulable objects, nonmanipulable objects, or animal), and their interaction term, together with random intercepts for participants and items, using the packages *lme4* (Version 1.1–31; Bates, Mächler, Bolker, & Walker, 2015) with the optimizers *bobyqa* and *nloptwrap*, and *lmerTest* (Version 3.1–3; Kuznetsova, Brockhoff, & Christensen, 2017) to retrieve *p*‐values in R (Version 4.2.2; R Core Team, 2022). To assess the statistical significance of the fixed factors and their interaction, we used likelihood‐ratio tests with χ^2^, comparing models with and without the factors and interaction of interest. For post‐hoc comparisons and to follow‐up interactions, we used *emmeans* with the Tukey adjustment (Version 1.8.2; Lenth, 2022). Data and analysis code are available at OSF.

### 3.1 Gesture

Based on the earlier rationale (see Introduction), the preregistration predicted an interaction between visual experience and semantic category in the frequency of gesture production and strategies. The hypotheses were: (1) blind participants would produce fewer gestures than sighted participants for non‐manipulable objects and animals but not for manipulable objects; (2) sighted participants would use the drawing strategy more than blind participants for non‐manipulable objects and animals, but not for manipulable objects; and (3) sighted participants would use the personification strategy more than blind participants for animals, but not manipulable and non‐manipulable objects.

Descriptive statistics for the total number of gestures across conditions and the average number of gestures per word are provided in Table S2. Sighted participants often produced multiple gestures for a single word, whereas blind participants predominantly produced only one gesture per trial in 64% of manipulable objects, 75% of non‐manipulable objects, and 75% of animals (see Table S3 for the percentages of trials depicted with one or more gestures). So, this contrast was most pronounced for animals, followed by non‐manipulable and manipulable objects.

#### 3.1.1 Frequency of gesture production

**Fig. 2:**
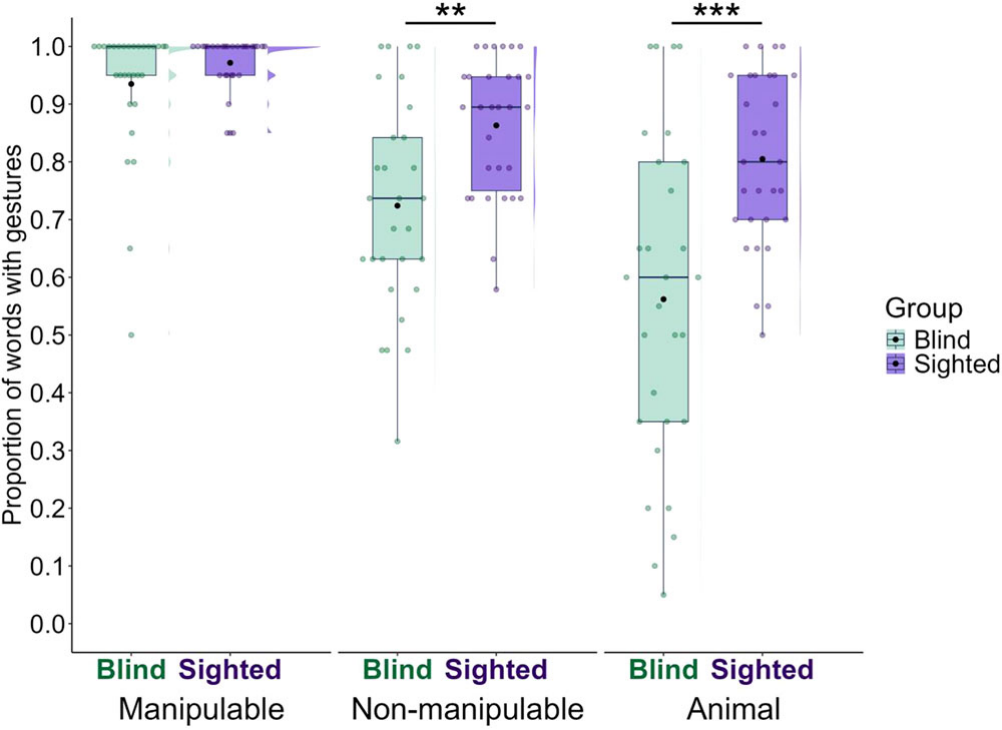
Proportion of words with gestures by group (blind vs. sighted) and semantic category (manipulable vs. nonmanipulable vs. animal). Colored dots represent each participant; black dots represent the group mean; half‐violin shapes show the distribution. Asterisks indicate the results of *emmeans* comparisons with Bonferroni correction, *p < .05; **p < .01; ***p < .001.

Following our preregistration, we compared whether blind and sighted participants differed in how often they produced at least one gesture per word across semantic categories (Fig. 2). A glmer model on binary values for gesture presence (0 = no, 1 = yes) as a dependent variable with fixed effects of visual status and semantic category revealed an effect of visual status, χ^2^ (1) = 11.03, *p* = .001. Overall, sighted participants produced gestures for more words than blind participants, β = 1.39, *SE* = .40, *z* = 3.52, *p* < .001. The model revealed an effect of semantic category, χ^2^ (2) = 57.34, *p* < .001, and also a significant interaction, χ^2^ (2) = 8.19, *p* = .017. As hypothesized in the preregistration, blind participants produced gestures for fewer words than sighted participants for non‐manipulable objects (β *=* −1.22, 95% CI = −2.04 to −.39, *SE* = .42, *z* = −2.90, *p* = .004) and animals (β = −1.64, 95% CI = −2.45 to −.84, *SE* = .41, *z* = −3.99, *p* < .001), but there was no significant difference between the groups for manipulable objects (β = −.66, 95% CI = −1.63 to .31, *SE* = .49, *z* = −1.34, *p* = .18).

#### 3.1.2 Frequency of gesture strategies

Next, as preregistered, we compared blind and sighted groups in terms of their gesture strategies for each semantic category. We analyzed the presence of each gesture strategy (0 = no, 1 = yes) with four preregistered glmer models, one for each gesture strategy (acting, drawing, personification, representing), with fixed effects of visual status and semantic category. We present data excluding skipped trials, but note that the results are the same when skipped trials are included.

**Fig. 3:**
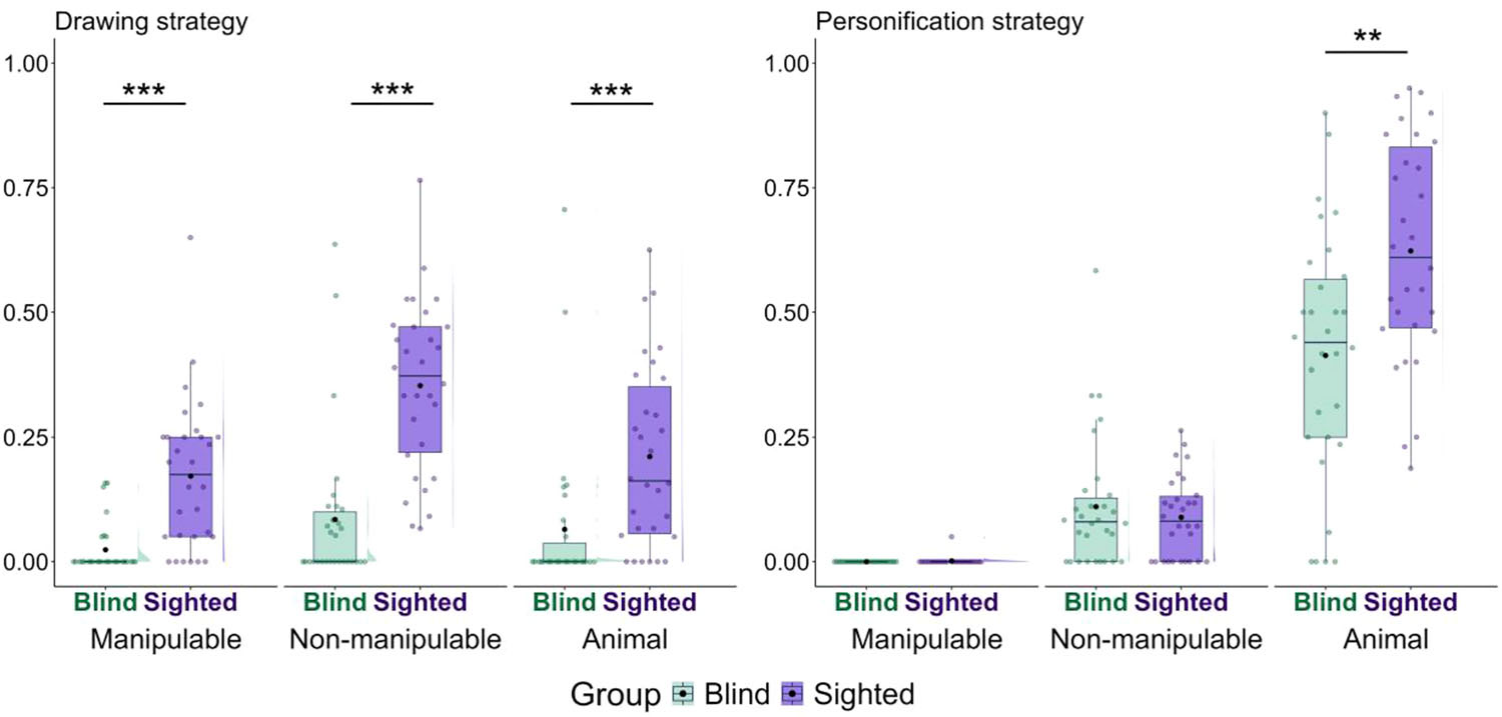
Proportion of words for which drawing and personification strategies were observed at least once. The proportion was calculated by dividing the total number of trials for which each strategy was produced by the total number of valid trials (i.e., excluding the skipped trials) per participant per semantic category. See Fig. 2 for the legend of symbols and colors.

We first assessed whether non‐manipulable objects and animal concepts elicited fewer drawing and personification strategies in the blind participants because of their different visual access (Fig. 3). Table 1 provides the model summaries and key findings for drawing and personification strategies.

**Table 1:** Model summaries and key findings for drawing and personification strategies, for which we predicted group differences based on visual status

Drawing strategy					
Effect	χ^2^ (df)	β	*SE*	*z*	*p*‐value
**Visual status**	35.51 (1)	−2.80	0.45	−6.23	< .001***
Blind participants produced four times fewer drawing gestures than sighted participants.					
**Semantic category**	6.88 (2)				.032*
non‐manipulable versus manipulable		1.33	.49	2.70	.019*
non‐manipulable versus animals		.82	.49	1.66	.22
manipulable versus animals		−.51	.49	1.04	.55
Participants produced about twice as many gestures with the drawing strategy for non‐manipulable than for manipulable objects.					
**Interaction**	9.32 (2)				.010**
manipulable		−3.31	.55	−6.01	< .001***
nonmanipulable		−2.97	.48	−6.16	< .001***
animals		−2.06	.51	−4.02	< .001***
Across all categories, blind participants used fewer gestures with the drawing strategy than sighted participants.					

Personification strategy^a^					
Effect	χ^2^ (df)	β	*SE*	*z*	*p*‐value
**Visual status**	3.17 (1)				.08
There was no significant difference between blind and sighted participants.					
**Semantic category**	38.01 (1)				< .001***
animals versus non‐manipulable		3.79	.50	7.60	< .001***
Participants produced roughly five times more gestures with the personification strategy for animals than for nonmanipulable objects.					
**Interaction**	12.58 (1)				< .001***
nonmanipulable		.20	.39	.53	.60
animals		−.98	.34	−2.85	.004**
Blind participants produced about 1.5 times fewer gestures with the personification strategy for animals than sighted participants but not for nonmanipulable objects.					

^a^Please note that due to the limited number of observations using this strategy in the manipulable objects category (resulting in a floor effect), we removed the manipulable objects category from the model to avoid producing a false interaction.

**Fig. 4:**
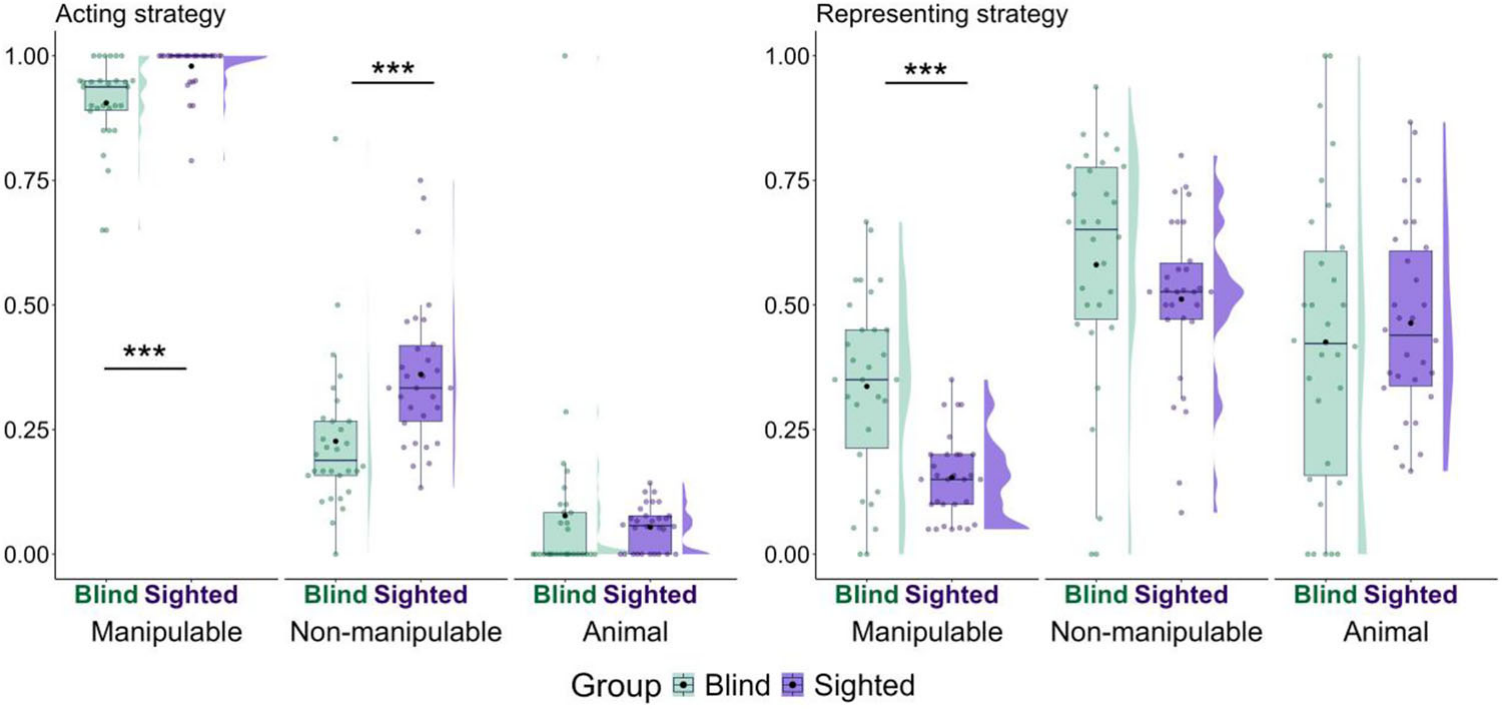
Proportion of words for which acting and representing strategies were observed at least once. See Fig. 2 for the legend of symbols and colors.

For the acting strategy, we predicted no group difference because there are no experiential differences across the blind and sighted groups here (Fig. 4). Finally, no theory makes predictions for the representing strategy, as Ortega and Özyürek (2020a) found that participants used this strategy frequently but not specifically for any semantic category (Fig. 4). Nonetheless, for completeness, analyses of this strategy were also conducted because it is one of the main gesture strategies and appears to be used by blind participants. Table 2 provides the model summaries and key findings for acting and representing strategies.

**Table 2:** Model summaries and key findings for acting and representing strategies, contrary to predictions of no group differences

Acting strategy					
Effect	χ^2^ (df)	β	*SE*	*z*	*p*‐value
**Visual status**	20.57 (1)	−1.28	0.26	−5.00	< .001***
Blind participants produced roughly 1.25 times fewer gestures with the acting strategy than sighted participants.					
**Semantic category**	75.53 (2)				< .001***
manipulable versus non‐manipulable		5.89	.88	6.71	< .001***
manipulable versus animals		10.11	1.1	9.50	< .001***
non‐manipulable versus animals		4.22	.98	4.31	< .001***
All participants used the acting strategy most for manipulable objects, roughly 3.5 times more than for nonmanipulable objects, and least for animals.					
**Interaction**	7.64 (2)				.022*
manipulable		−1.83	.41	−4.48	< .001***
nonmanipulable		−1.35	.32	−4.21	< .001***
animals		−0.37	.45	−0.82	.41
Blind participants produced roughly 1.5 times fewer gestures with the acting strategy than sighted participants for manipulable and no‐nmanipulable objects.					

Representing strategy					
Effect	χ^2^ (df)	β	*SE*	*z*	*p*‐value
**Visual status**	5.48 (1)	0.59	0.25	2.39	.017*
Blind participants produced roughly 1.2 times more gestures with the representing strategy than sighted participants.					
**Semantic category**	18.39 (2)				< .001***
animals versus manipulable		1.51	.47	3.23	.004**
animals versus nonmanipulable		−.58	.47	−1.23	.43
non‐manipulable versus manipulable		2.09	.47	4.40	< .001***
All participants used the representing strategy roughly two times more for animals and nonmanipulable objects than for manipulable objects.					
**Interaction**	29.03 (2)				< .001***
manipulable		1.23	.28	4.39	< .001***
non‐manipulable		.50	.28	1.77	.076
animals		−.07	.29	−.25	.80
Blind participants produced roughly twice as many gestures with the representing strategy as sighted participants for manipulable objects, but not for non‐manipulable objects or animals.					

#### 3.1.3 Summary

As expected, blind participants produced substantially fewer gestures overall than sighted participants, and this difference was especially pronounced for concepts that rely heavily on visual information (non‐manipulable objects and animals), but not for concepts that rely more on motor information (manipulable objects). Exploratory analyses (Supplementary S9) further showed that when both blind and sighted participants gestured, they tended to favor similar strategies: both groups used the acting strategy most often for manipulable objects, the representing strategy for non‐manipulable objects, and the personification and representing strategies for animals. Nonetheless, there were still nuanced differences in how often particular strategies appeared—for instance, blind participants produced fewer gestures overall with drawing (across all categories) and personification strategies (for animals) than sighted participants—suggesting that visual experience may shape not only gesture frequency but also aspects of strategy selection.

### 3.2 Features

We analyzed the feature data^3^ similarly to the gesture data. Based on the earlier rationale (see Introduction), we predicted an interaction between visual experience and semantic category in the frequency with which perceptual and non‐perceptual features would be listed. The hypotheses were: (1) blind participants would produce fewer perceptual features of concepts than sighted participants; (2) blind participants would produce more non‐perceptual features of concepts than sighted participants; and (3) the difference between blind and sighted participants for hypotheses 1 and 2 would be bigger for the non‐manipulable objects and animals than manipulable objects.

#### 3.2.1 Frequency of perceptual and non‐perceptual features

**Fig. 5:**
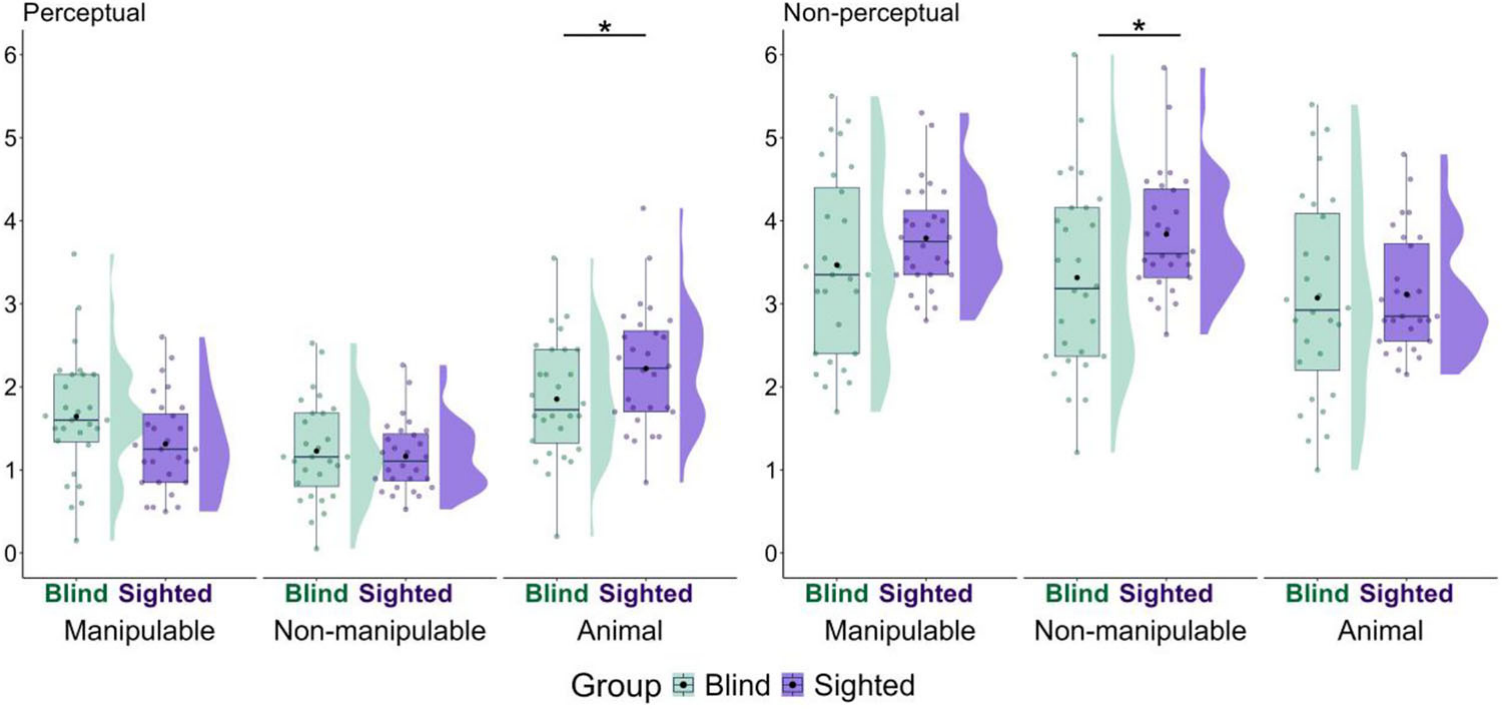
Mean count of perceptual and nonperceptual features listed by semantic category and group. See Fig. 2 for the legend of symbols and colors.

We first compared whether blind and sighted participants differed in the number of perceptual features they listed (Fig. 5). Against our hypothesis, the model did not reveal an effect of visual status, χ^2^ (1) = .003, *p* = .96, but did show an effect of semantic category, χ^2^ (2) = 21.99, *p* < .001. There was, however, an interaction between visual status and semantic category, χ^2^ (2) = 61.66, *p* < .001. Compared to sighted participants, blind participants produced fewer perceptual features for animals (β = −.37, 95% CI = −.70 to .04, *SE* = .17, *t* = −2.21, *p* = .031) but not for manipulable objects (β = .33, 95% CI = −.001 to .66, *SE* = .17, *t* = 1.98, *p* = .051). There was no group difference for non‐manipulable objects (β = .06, 95% CI = −.27 to .40, *SE* = .17, *t* = .38, *p* = .70).

We then compared whether blind and sighted participants differed in the number of non‐perceptual features they produced among all features (Fig. 5). The model did not reveal an effect of visual status, χ^2^ (1) = 1.51, *p* = .22, but did reveal an effect of semantic category, χ^2^ (2) = 11.30, *p* = .004. There was, however, an interaction between visual status and semantic category, χ^2^ (2) = 19.60, *p* < .001. Surprisingly, blind participants produced fewer non‐perceptual features than sighted participants for non‐manipulable objects (β = −.52, 95% CI = −1.02 to −.03, *SE* = .25, *t* = −2.1, *p* = .040), but not for manipulable objects (β = −.32, 95% CI = −.82 to .17, *SE* = .25, *t* = −1.30, *p* = .20) or animals (β = −.04, 95% CI = −.54 to .45, *SE* = .25, *t* = −.17, *p* = .86).

#### 3.2.2 Exploratory analyses: Subcategories of perceptual and nonperceptual features

**Fig. 6:**
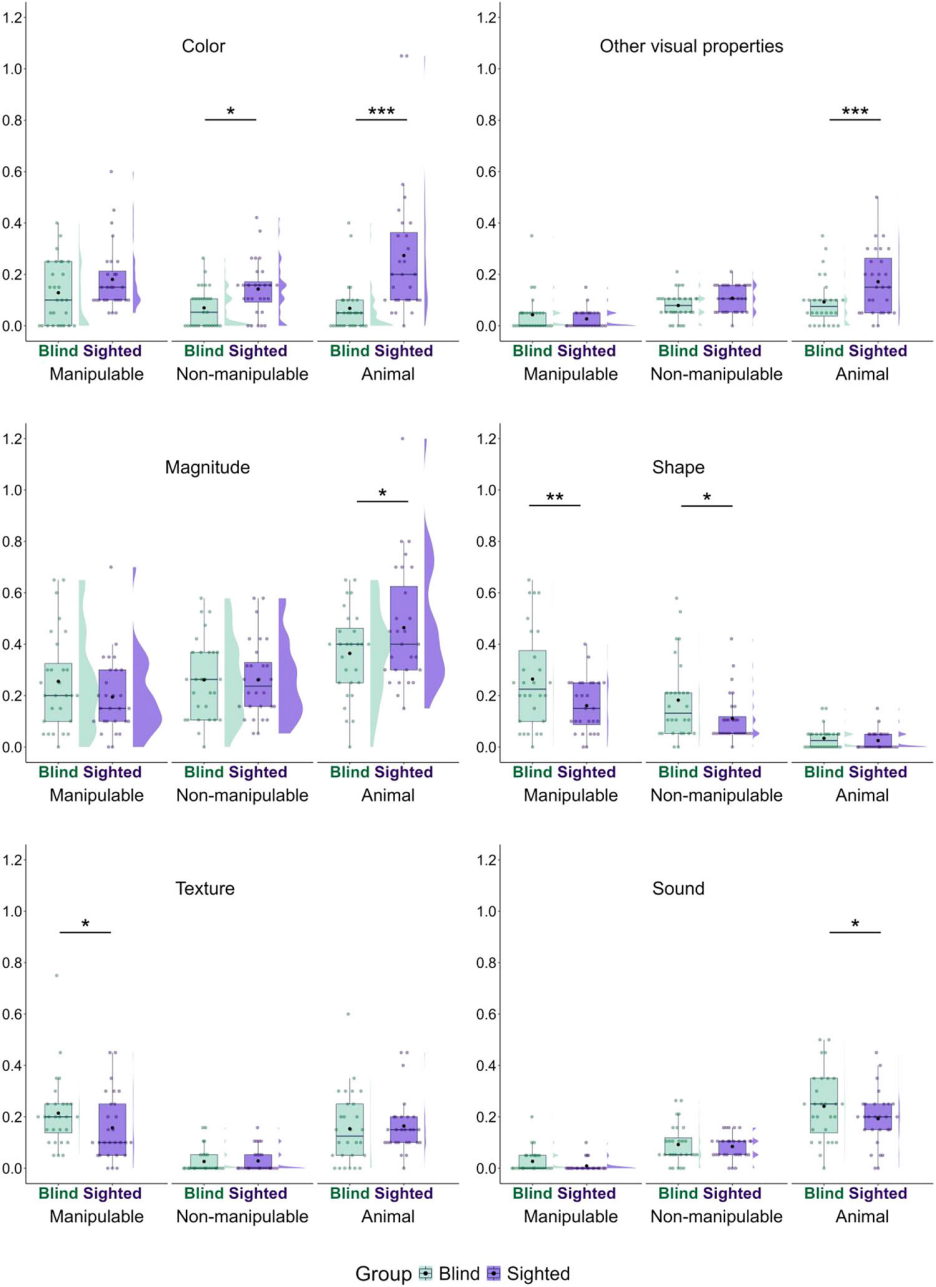
Mean count of perceptual feature subcategories by semantic category and participant group. See Fig. 2 for the legend of symbols and colors.

We further explored whether blind and sighted participants differed in terms of the specific perceptual features they reported among all features (Fig. 6). There were no significant differences in the non‐visual properties of taste, smell, or temperature, nor were there differences in parts or kinematic movement. However, blind participants listed roughly 1.5 times fewer visual features, such as the color of non‐manipulable objects and animals, other visual properties (e.g., shiny), and the magnitude (e.g., tall) of animals, but roughly 1.2 times more features related to the shape of both manipulable and non‐manipulable objects, the texture of manipulable objects, and animals sound. We also conducted exploratory analyses for different types of non‐perceptual features (i.e., functional, taxonomic, and encyclopedic). Blind and sighted participants did not differ in the number of functional and taxonomic features produced in any semantic category, but blind participants produced fewer encyclopedic features than sighted participants for non‐manipulable objects (see Supplementary S10 and S11 for model summaries of perceptual and non‐perceptual features).

## 4 Discussion

In this preregistered study, we investigated whether blind and sighted people behave differently in gestural and verbal responses for words that rely primarily on visual (non‐manipulable objects and animals) versus motor information (manipulable objects). The results show that blind people share substantial knowledge with sighted people across these categories, but differ in the fine‐grained details aligned with their own sensorimotor experience, with the most pronounced differences observed in animal concepts.

In silent gestures, as predicted, blind people produced gestures relatively less often than sighted people for non‐manipulable objects and animals, but did not differ for manipulable objects. We also observed that sighted participants often produced multiple gestures to represent a single concept, whereas blind participants typically produced only one. This pattern held even for manipulable objects. For example, for the concept *banana*, both blind and sighted participants might start by imitating peeling it. However, sighted participants were more likely to expand on this by also imitating eating the banana, outlining its shape, or even combining all three gestures in sequence. It is unclear why sighted people behave in this way, but blind people do not.

In the feature listing task, we found that compared to sighted participants, blind participants produced fewer perceptual features for animals and fewer non‐perceptual features for non‐manipulable objects. The exploratory analyses of perceptual features further revealed that blind participants mentioned animal sounds more frequently but reported color, visual properties (e.g., shiny), and size (e.g., tall, big) less often than sighted participants. Despite these differences in feature frequency, there was substantial overlap between groups in the types of perceptual and non‐perceptual features listed. Therefore, the prediction that blind participants would produce fewer perceptual features for both non‐manipulable objects and animals was only supported for the animal category.

Looking across tasks, we found that blind people mentioned object shapes more than sighted people for non‐manipulable objects in the feature listing task. However, they produced fewer gestures with the drawing strategy for non‐manipulable objects, although this strategy relies on the objects’ overall shape (i.e., outlining the contours of objects). This suggests that analog knowledge derived from perceptual experiences about the contours of large objects might differ from categorical knowledge about object shapes learned through language (e.g., Landau & Jackendoff, 1993).

Unlike color and appearance, object shape and size are not exclusively visual properties and can be perceived through touch too (Lee Masson, Bulthé, Op de Beeck, & Wallraven, 2016). Therefore, one might expect to find comparable knowledge between blind and sighted people regarding the size and shape of manipulable objects (e.g., Peelen et al., 2013, 2014; Xu et al., 2023). However, blind people mentioned the shape of non‐manipulable objects more frequently than sighted people, even though this was not predicted because of the limited motor affordances of those objects. This suggests that knowledge acquired through language may contribute to learning certain attributes about some distal concepts (e.g., the shape of non‐manipulable objects) but not others (e.g., the size of animals). This difference could be attributed to the level of familiarity with different sorts of entities. Kim et al. (2019), for example, found that blind participants rated animals as less familiar than sighted participants. Our blind participants also confirmed this anecdotally. At the end of the experiment, blind participants mentioned that animal concepts were particularly challenging for them due to their lack of familiarity with the movements of certain animals.

Blind people also produced fewer gestures with the personification strategy for animal concepts (Ortega & Özyürek, 2020a; van Nispen et al., 2017). The personification strategy relies on mapping visual and kinematic features (i.e., how certain body parts move) of non‐human animate entities onto the body. This suggests that analog representations of visuospatial aspects of animals, including kinematic movements, are less readily available to blind people. The feature data provided converging evidence of this, too. Blind people produced fewer color, visual properties, and size‐related perceptual features for animals than sighted people. Furthermore, although sighted people frequently reported specific colors (e.g., *yellow lion*, *red cockscomb*) in association with concepts, blind people only mentioned that an object was *colorful* in a general sense. Consistent with previous findings (Kim et al., 2019; Tian et al., 2024), the present results indicate that blind and sighted people diverge in specific knowledge of animal appearance, such as size, height, and color. Moreover, the results from gesture contribute to the literature by illustrating this divergence through analog representations of visuospatial characteristics, including kinematic movements.

Differences in the frequency of gesture strategies across groups highlight adaptations to individuals’ unique sensorimotor experiences. Another noteworthy aspect is that although the acting strategy was the most preferred for manipulable objects in both blind and sighted groups, blind people unexpectedly used the representing strategy more than sighted people for manipulable objects. To depict the concept *spoon*, for example, sighted people imitated eating with a spoon (acting strategy). In contrast, blind people showed the concave shape of a spoon (i.e., a shape that curves inwards) with their hands (representing strategy). The representing strategy might be more accessible to blind people because of their salient tactile experiences with objects, which might be as important as motor experience. Blind people also used the acting and representing strategies simultaneously in 15% of their gestures depicting manipulable objects. Tactile and motor experiences are intertwined in daily life. However, sighted people do not typically integrate them together in one gesture, indicating that tactile experience with objects is more prominent for blind people than for sighted people. Blind people also mentioned the texture and shape of manipulable objects more frequently than sighted people in the feature listing task, which corresponds with their greater use of the representing strategy in gestures. Thus, these findings suggest that while perceptual features and gestural forms for concepts may be broadly similar across blind and sighted people, sensorimotor experiences fine‐tune the gestures, aligning with earlier research indicating that gestures reflect sensorimotor experiences with objects (Beilock & Goldin‐Meadow, 2010; Cook & Tanenhaus, 2009; Goldin‐Meadow & Beilock, 2010; Hostetter & Alibali, 2008, 2019; Pouw et al., 2020).

Surprisingly, despite these subtle differences in the frequency of gesture strategies, the general tendency for the strategies used for specific semantic categories was similar across groups. Previous studies have shown that sensorimotor associations of concepts systematically influence the use of gesture strategies in sighted people (Masson‐Carro et al., 2016, 2017; Ortega & Özyürek, 2020a, 2020b; van Nispen et al., 2017). Here, we found that when they gestured, blind people were similar to sighted people in which strategy they used most often for each semantic category—that is, the acting strategy for manipulable objects, the representing strategy for non‐manipulable objects, and personification and representing strategies for animals. So, although blind people produced fewer gestures using the personification strategy than sighted people, it remained their preferred strategy for animal concepts, alongside the representing strategy. This shared pattern also aligns with previous findings showing regularities in gesture strategies across sighted people for different semantic categories (Ortega & Özyürek, 2020a, 2020b; van Nispen et al., 2017). Ortega and Özyürek (2020b) found these preferred strategies for manipulable objects and animals in Dutch and Mexican Spanish, and we replicate them here in Turkish, an unrelated sociocultural and linguistic context. However, for non‐manipulable objects, Turkish sighted speakers mostly preferred the representing strategy, in contrast to the drawing strategy preferred by Dutch and Mexican Spanish speakers in Ortega and Özyürek (2020b). This suggests there are both universal and cultural patterns for mapping semantic categories to visual‐manual expressions.

It is important to acknowledge that visual experience may indirectly shape individuals’ sociocultural and linguistic experiences. One could argue that differences in blind people's gesture use might be attributed to their lack of exposure to others’ gestures rather than reflecting differences in conceptual representations. Even if blind people have similar conceptual representations to sighted people, they still need to transform that knowledge into gestures. People often understand what a newly constructed gesture may mean thanks to iconicity (i.e., similarity between the form and the meaning of a referent; Perniss, Thompson, & Vigliocco, 2010; Taub, 2001) and also to systematicity in the forms of gestures people produce (van Nispen et al., 2017). This gestural experience may ultimately drive the systematicity in silent gestures and, therefore, increase the transparency of gestures (see also van Nispen et al., 2017). It has been shown that success in the interpretation of gestures is greater for sighted than blind gesturers (Fay et al., 2022). This suggests that visual experience might also affect gesture production through differences in communicative and sociocultural experiences. While further research should explore this possibility, the preferred strategies across semantic categories shared between blind and sighted people (e.g., the predominant use of the acting strategy for manipulable objects) indicate that not all results can be attributed to the lack of gestural experience alone. Instead, this suggests an influence of sensorimotor experiences on analog expressions of conceptual representations.

In the present study, we did not measure the systematicity and comprehensibility (i.e., how easily others can interpret their meaning) of gestures. Systematicity involves evaluating whether gestures are similar not only in the strategy used but also in hand configuration, orientation, movement, and placement (see Gerardo & Özyürek, 2020a). We observed that even when both blind and sighted people use the same gestural strategy for depicting a concept, the content of the gesture might differ. For example, two participants produced an acting strategy to depict the concept *bread*, but the sighted gesturer imitated cutting bread, whereas the blind gesturer imitated smelling bread. So, although both participants rely on their sensorimotor experiences with bread, it appears that the olfactory experience of the bread's smell is more prominent for the blind participant than for the sighted participant, who relies on the motor experience of cutting bread. A similar pattern may also be observed in the gestures of sighted individuals across cultures. While universal tendencies in gesture strategy preferences exist (e.g., the acting strategy used for manipulable objects by Dutch, Mexican Spanish, and Turkish speakers), the systematicity of gestures may vary across cultures. This suggests that further investigation of gesture forms may be a fruitful line of inquiry for examining how sociocultural, linguistic, and sensorimotor experiences shape conceptual representations.

## 5 Conclusion

The current study contributes to the ongoing debate about how the source of knowledge influences the structure of conceptual representations. Previous research suggests that blind people gain substantial sensorimotor knowledge about concepts through indirect experiences mediated by language, as reflected in their semantic judgments, which often closely align with those of sighted people (e.g., Bedny et al., 2019; Bedny & Saxe, 2012; Bi, 2021; Kim et al., 2021; Landau & Gleitman, 1985; Lewis et al., 2019; Marmor, 1978; Saysani et al., 2018, 2021). However, some studies have revealed qualitative differences in how blind and sighted people use conceptual knowledge, suggesting that visual experience shapes the organization of their representations (e.g., Connolly et al., 2007; Kim et al., 2019; Lenci et al., 2013; Shepard & Cooper, 1992; Tian et al., 2024). Here, we provide evidence that visual experience shapes, to some extent, how concepts are expressed in gestures and words. Blind people have broad conceptual knowledge largely similar to that of sighted people, but they differ in finer details that reflect their sensory experiences. Overall, these findings reveal nuanced differences in certain aspects of how concepts are communicated, emphasizing the interplay of visual experience and conceptual representation.

## Notes

1 Pointing gestures were coded only for complementary purposes as we followed Ortega and Özyürek (2020a). As this category was not the main interest of the present study, the results are presented as Supplementary Data (see Supplementary S6).

2 In our preregistered analysis plan, we intended to conduct exploratory analyses to explore group differences in types of perceptual features listed. The final subcategories were determined based on the observed data.

3 Two blind participants did not complete the feature listing questionnaire, so their matched sighted controls were also excluded; including them did not change the results.
